# Intersystem Implications of the Developmental Origins of Health and Disease: Advancing Health Promotion in the 21st Century

**DOI:** 10.3390/healthcare4030045

**Published:** 2016-07-15

**Authors:** Michael D. Barnes, Thomas L. Heaton, Michael C. Goates, Justin M. Packer

**Affiliations:** 1 Department of Health Science, Brigham Young University, Provo, UT 84602, USA; tomlheaton@gmail.com (T.L.H.); justin.packer1@gmail.com (J.M.P.); 2 Harold B. Lee Library, Brigham Young University, Provo, UT 84602, USA; michael_goates@byu.edu

**Keywords:** DOHaD, developmental origins of health and disease, life course theory, LCT, social determinants of health, public health, health promotion, health policy, health systems, research translation

## Abstract

The developmental origins of health and disease (DOHaD) theory and life course theory (LCT) are emerging fields of research that have significant implications for the public health and health promotion professions. Using a DOHaD/LCT perspective, social determinants of health (SDH) take on new critical meaning by which health promotion professionals can implement DOHaD/LCT guided interventions, including recommended policies. Through these interventions, public health could further address the sources of worldwide chronic disease epidemics and reduce such disease rates substantially if related policy, programs, and interdisciplinary and multi-sector collaboration are emphasized. Additional characteristics of the most effective interventions involve context-specific adaptation and societal structures that impact upstream, early life environments on a broad scale, influencing multiple locations and/or diseases.

## 1. Introduction

A rapidly expanding body of clinical and biological science suggests that adult health and disease may originate in utero, indicating that prenatal conditions (in addition to influences later in life) may have health-related consequences in adulthood. This life course concept has several names, but is often described as the developmental origins of health and disease (DOHaD) or as life course theory (LCT). As the rapidly expanding knowledge base about this new science emerges, public health practitioners, researchers, and academicians need to consider how vulnerability to most chronic diseases (e.g., heart disease, stroke, diabetes, obesity) [1] and other health foundations (e.g., cognitive functioning, mental well-being) may further shape current understanding of the roots of these diseases, including their prevention. Regardless, it is clear that all entities within public health, including health promotion, should consider the program, intervention, and policy implications of DOHaD and LCT.

The purpose of this paper is to describe the implications of DOHaD/LCT on public health. Focused as a proof of concept for health promotion and disease prevention, this paper will specifically consider DOHaD’s potential impact on select interests including social determinants of health (SDH), public health, population health, health promotion, health policy, and health systems.

## 2. DOHaD/LCT Definitions

Before describing the implications of DOHaD to public health, population health, SDH, health promotion, health policy, and health systems, it is important to consider the most widely accepted terms used by clinical and biologic scholars. These definitions include aspects involving the First Thousand Days, the Barker Hypothesis, Developmental Plasticity, Epigenetics, the Developmental Origins of Health and Disease, and Life Course Theory. It is important to acknowledge that DOHaD and LCT each add subtle perspectives, which are defined later in this section. For simplicity, however, we will, hereafter, use the more generic term DOHaD to reference both aspects.

### 2.1. First Thousand Days

Researchers who focus their attention on the embryo, fetus, or newborn refer to this critical time as the First Thousand Days. The First Thousand Days is a critical phase when a large fraction of adult health is programmed [2]. This phase begins at conception and generally extends to age two and represents an opportunity for carefully targeted interventions that may have significant impacts [3].

### 2.2. Barker Hypothesis

Among the fundamental concepts in the field, the Barker Hypothesis has persisted and evolved as research has been conducted over the past few years. The enduring concept is that prenatal factors affect disease and health status later in life. The latest of this iterative theory involves predictive adaptive response (PAR) [4]. According to the theory, genes are promoted or repressed to adapt to expected future environments. These anticipated future environments are shaped by exposure to the current environment, especially when developmental plasticity is most operative (see “Developmental Plasticity”). If inappropriate PARs are made to meet a vulnerable time, the risk of disease in later life appears to be greater [5]. For example, “insulin resistance can be advantageous before and after birth in poor nutritional environments but it also creates a disease risk in the future according to the environment the child/adult will be living in” [4]. Further, since the timing of effects vary for different organs, long term exposure to poor environments may widen the impact of disease risk later in life [4].

### 2.3. Developmental Plasticity

Developmental Plasticity (DP) is a key foundation to understanding DOHaD because it describes the early conditioning mechanism by which one genotype can give rise to a range of phenotypes allowing the organism to respond to environmental cues, often with an adaptive advantage [6]. Healthy development is believed to be an adaptive process that has been engendered to promote resilience and plasticity in the face of changing and often constraining environmental contexts, but such plasticity declines with age [7]. This phenomenon is closely related to epigenetic modification wherein genes are promoted or repressed in the presence of existing conditions.

### 2.4. Epigenetics

Epigenetic changes are modifications to DNA that result in or control gene expression and thus function. All cells have the same genes. However, as cells differentiate into unique types of cells during development each new cell type has a specific set of genes turn on or off through the epigenetic system. Of interest for this paper, sometimes certain environmental, nutritional or stress conditions, or chemicals, can interfere with this differentiation process and produce cells with some genes turned on or off inappropriately. This leads to increased susceptibility to disease across the lifespan. Such gene expression may involve DNA methylation, histone acetylation, or small non-coding RNAs, and it offers important insight into the development of obesity, insulin resistance, and hypertension [4,5]. Such genetic expression can be passed on to the next generation, but is not explained by changes in the chromosomal system or DNA sequence [5,8,9]. Moreover, because epigenetic reprogramming occurs during folliculogenesis and embryogenesis, any disturbance of the normal natural environment during these critical phases could cause epigenetic alterations, an excess of which could potentially cause pre- or postnatal death [10].

### 2.5. Life Course Theory

Life Course Theory asserts that non-communicable diseases (NCDs) are developed gradually over a lifetime [7] given social, economic, and environmental patterns that are closely linked to community, neighborhood, and household circumstances. As a result, LCT is population focused, and is rooted in social determinants and social equity models [11]. Similar to other related DOHaD concepts, LCT notes the influence of early exposure on future outcomes, that they occur during sensitive periods through broad physical and social factors, and that “inequality in health reflects more than genetics and personal choice” [11]. Future research should drive the development of a life course model of aging that integrates specific diseases or clinical conditions, functional aging and well-being, and should include socioeconomic characteristics [12].

### 2.6. Developmental Origins of Health and Disease

DOHaD is the consequence of fetal responses to its environment [5,13]. More specifically, DOHaD explains that “environmental exposures during critical periods of development may cause subtle changes in certain biological functions, although practically invisible, and can increase the risk of disease and dysfunction later in life” [14]. As such, “DOHaD research shifts the focus upstream to how environmental factors experienced by the developing fetus impact later development of adult health or disease” [15]. For example, chronic stress has been associated with enhanced visceral fat deposition and increased incidences of metabolic diseases later in life [16]. Maternal smoking is another fetal stressor which has been linked to the subsequent development of obesity and disease throughout life. Taken together, these findings have led to the discovery of DOHaD [17].

## 3. Social Determinants of Health and DOHaD

### 3.1. Interconnection of Environmental, Genetic, and Developmental Influences

Over the past several decades, the SDH have played a substantial role in influencing current public health practice and policy. Under a SDH framework, health inequities—preventable and unjust health status differences—are the product of the social and environmental conditions in which people live and work [18]. Using a social justice perspective, SDH researchers and practitioners target specific social conditions (e.g., housing, employment, education, etc.) to understand health disparities between groups and to develop more effective interventions and policy recommendations. In many ways, the perspectives and goals of a social determinants framework are similar to DOHaD. While SDH covers all age groups, DOHaD is more narrowly focused on key periods of early human development. In essence, DOHaD can be viewed as a more specific element of the SDH framework, focusing on a narrower time period in an individual’s development. Wallack and Thornburg [1] clearly place DOHaD in the realm of SDH when they assert that “developmental origins is the ultimate social and health equity lens because it helps us understand how life history, sociology, and biology combine to create lifelong prospects for health and social success at the earliest stages.” Likewise, DOHaD draws upon social, economic, and environmental drivers for health and disease within social determinants and social equity models [11]. As a result, DOHaD scholars urge public health workers “to identify and address root causes of disparities at the population level” [11].

Under both DOHaD and SDH perspectives, there are strong connections between social, environmental, genetic, and developmental influences [9]. Regarding a life course perspective, a negatively synergistic relationship exists between genetics, epigenetics, and social determinants, leading to poorer health outcomes and social exclusion [19]. The relationship between health determinants, genetics, and epigenetic influences is particularly relevant from a developmental origins perspective where early exposure to epigenetic and environmental influences can have an inordinate impact on health outcomes throughout the life course [20].

### 3.2. Social Environment Impact on Health

From an SDH standpoint, the social environment has a profound impact on human health. For example, chronic diseases cannot be fully understood without considering the social environment contributing to their existence [21]. As an important epigenetic influence, the social environment alters gene expression and disease occurrence. This knowledge is drastically changing current understanding about the dynamic manner in which epigenetics control intergenerational inheritance of natural traits [19]. The mediating influence of the social environment on gene expression and health status is particularly powerful during early developmental stages. The socioeconomic environment experienced during childhood has a more profound influence on adult health and disease than at any other time of life [2,22,23].

### 3.3. Social Determinants, Stress, and DOHaD

It is important to consider the levels of stress and hardship experienced at various stages of development. Early-life adversity and stress have long-term health implications for individuals and communities [24]. Stress and hardship create less stable home and family environments, resulting in poorer health outcomes for children [25]. Stress is influenced by a wide variety of conditions, such as poverty, employment opportunities, nutritional deficiencies, level of education, and other financial deprivations and hardships [1,3,19,23]. For example, unequal access to education has multigenerational impacts on employment, financial, and health trajectories. Poverty not only affects the health and well-being of young children directly, but it also influences the intrauterine environment for developing fetuses. Of particular interest is the impact of financial deprivation on maternal stress, which in turn has negative generational health impacts [15,26].

## 4. Implications of DOHaD on Public Health, Population Health, and Health Promotion

### 4.1. Emerging Public Health Functions

Public health functions exert influence through interventions or policies that have both upstream (structural) and downstream (environmental) factors [21]. Within these factors, public health functions exert their influence on NCD rates. The resulting trajectories of health related NCDs are synergistic, diverge over time, and reveal the disparities in health that parallel socioeconomic statuses [27]. These emergent disparities in socioeconomic status and health show that public health investment in early life has the potential for instigating substantial disparity improvement [7]. Given that expectation, thorough implementation of DOHaD research offers public health decision makers opportunities to significantly influence population health [28]. These findings indicate that public health functions will have a substantial role in combating NCDs in collaboration with governments, communities, and researchers.

Currently, there are many public health concerns with NCDs worldwide that could be significantly ameliorated by applying the DOHaD paradigm. As it is more efficient and equitable to prevent diseases than to simply cure them [19], using the DOHaD theory to fight growing rates of NCDs could be an emerging, vital public health function that produces sizable societal and economic benefits. For example, NCDs have a greater economic impact than infectious diseases, afflict ten times as many people as HIV/AIDS, and account for 35 million deaths per year or 60% of all deaths globally [7,9]. As DOHaD research implementation has suggested effectiveness against NCDs [13,29,30], these global statistics represent an extraordinary opportunity for public health workers to contribute to the alleviation of the burdens of such diseases. In support of reaching this potential, the ecobiodevelopmental model suggests that public health functions should promote healthy societal and physical environments that will affect both the biology (through epigenetics) and the health and development (through life course sciences) of populations [26].

### 4.2. Link Population Health to Improved Service Subsystems

Many working in DOHaD have called for a health development system that expands across preventive health and clinical health sectors among traditional and nontraditional partners to support the full range of prevention, health promotion, and health optimization activities [25] for groups of people or populations. Public health’s role in those efforts should facilitate “anticipatory, early and preemptive interventions that are designed to build health capital, improve health development trajectories, and avoid future threats to optimal health outcomes” [31]. Such approaches require more collaboration and networking of physical and behavioral health and also include social, communal, and educational assets and resources [25].

Public health and medical related funding models may need to be refined away from “disease-by-disease funding [to better] address common causal pathways across conditions” [11]. There also needs to be a shift from discrete and episodic services toward “integrated, multi-sector service systems that become lifelong ‘pipelines’ for healthy development” [11]. Such funding and priority shifting provided from DOHaD could help adjust service delivery that would otherwise result in missed opportunities and an inefficient use of resources.

### 4.3. A Closer Look at Disease Prevention through Upstream Thinking

Support for public health entities to apply a DOHaD paradigm to chronic disease prevention is continually increasing. For example, researchers have identified prevention as the most effective approach to attain active and healthy aging [32]. Similarly, others have expressed a need for DOHaD to focus on chronic disease prevention by emphasizing early development [13,29,30] and primordial prevention [33]. Early health investments both maximize positive health and prevent disease [27]. Such viewpoints suggest that public health disease prevention initiatives that build on a DOHaD foundation will likely decrease rates of chronic diseases in needful ways.

Under an SDH framework, interventions to promote individual and community health should be targeted upstream to have the greatest impact. Upstream interventions are preventive in nature and attempt to address structural challenges that could potentially lead to adverse health outcomes [21]. For example, interventions to reduce social inequities at birth are likely to have a profound and widespread positive impact on health greater than efforts to enhance individualized health care later in life [19]. Part of this upstream focus should be applied to primordial, prenatal and early life interventions. Focusing primordial and primary prevention and environmental interventions at early life stages under a life course approach will provide better means of controlling human exposure to causative substances and reduce the burden of NCDs [29,33]. This focus on early life stages is a critical upstream intervention strategy to prevent negative health outcomes later in life. However, upstream interventions can go beyond the prenatal and early childhood timeframe. Indeed, social determinants impact the parental epigenome, which is subsequently inherited by offspring [19]. As epigenetic influences span multiple generations, it is likewise critical to adapt interventions accordingly.

### 4.4. Expanded Research Functions

While DOHaD becomes more confirmed in clinical and bench science professions, the need for discipline-specific research in public health is also needed. Like in other professions (e.g., nursing), there is insufficient DOHaD research [15]. To consider the research needs for public health, we will explore recommendations posed by DOHaD scholars. We will first address the suggested topics of interest and will then identify suggested research methods and procedures that are recommended for public health.

Suggested research issues below may deserve the greatest attention, and in most cases, provide a base upon which future study in public health can build. For example, there is an inadequate amount of research regarding the developmental origin of two global epidemics, including type-2 diabetes and obesity [12]. Further, nutrition practices and outcomes also have developmental implications on chronic disease and aging but need to be studied further [12]. Implementing research of the determinants of active and healthy aging could reveal better disease prevention strategies and reduce societal costs of the aging population [12].

Other recommended research topics involve genetic predisposition and epigenetic monitoring. For example, there is a key knowledge gap identifying tools to monitor epigenetic settings, related developmental trajectories and disease reduction [5]. That gap is emphasized by an ongoing need to study gene-environment interactions that integrate genetic predispositions, perinatal epigenetic, and functional teratogenesis to better understand pathways to primary and primordial prevention [33,34].

To widely apply DOHaD’s epigenetic mechanisms, an organizing framework or conceptual model may be needed to guide integrated efforts between multidisciplinary research, practice, and policy development [1]. Such a conceptual model/graphic needs to (1) integrate policies, programs and services that promote health over the life span and throughout generations; (2) reflect efforts over time, within the health sector and across other sectors; and (3) address the most fundamental factors that cause health disparities [11]. Additionally, a conceptual model should illustrate the connection between population health and developmental health at the individual level [31], and should frame health development positively through prevention and not entirely from the deficit or remediation angle [25]. Thus, a new or modified conceptual model will help promote DOHaD among various population health stakeholders.

Similarly, public health should test its assumption about the role of lifestyle and behaviors in the context of DOHaD [17,25]. For example, public health risks may no longer be mostly based on the assumption that personal choice (e.g., quantity and kind of foods eaten, physical inactivity, etc.) is the key factor for changing health outcomes [17]. Rather it must also consider additional complex events as contributing factors to the chronic disease epidemic, such as environmental exposure during early development. Translating DOHaD to public health may emphasize important research methods or procedures. Among those factors, promoting wide-scale translation involves the need for large longitudinal birth cohort studies with prospective measures of broad domains of exposures and outcomes [4,12,35]. This research should reflect multi-sector collaboration [29] to collectively understand the causal mechanisms and determine the best interventions [36]. Though ambitious, public health leaders could assert their interests for various funding sources (government, private, NGO, etc.) to support these types of research studies.

To prevent chronic disease under the DOHaD paradigm, public health personnel may use epigenetic determinants or epigenetic profiling research as guidelines to improve public health [13,33,37]. In epidemiological research, there is substantial evidence revealing epigenetic determinants as a link between childhood experiences and unhealthy conditions throughout later life [38]. Application of such research will require interdisciplinary collaboration to understand disease causal mechanisms and design novel, context-specific interventions [12,36]. By promoting such collaboration, the public health field will further fulfill its function to prevent chronic disease.

### 4.5. Expanded Risk Factor Reduction for Individual and Population Health

Public risk factor control is another public health function related to the implementation of DOHaD research. A DOHaD perspective has led many professional organizations to demand action against adverse environmental factors in order to protect the health of developing individuals [39]. New research suggests that the adverse effects of environmental pollutants on pregnant mothers, young children, and even fathers could influence the disease susceptibility of individuals who are at early developmental periods, though many questions remain about the pathways and extent of such effects [15]. Public health services that could reduce disease risk factors for maternal and child health include health education for adolescent girls and women concerning nutrition, exercise, weight management, emotional health, and social injustice advocacy. Additional services could focus on reducing poverty, crime, and racism [15]. Preventing disease risk factors and promoting healthy lifestyles could make a significant impact when applied from infancy [40]. Therefore, applying DOHaD research to inform these services could promote healthier environments and lifestyles, reduce NCD risk factors and strengthen prevention efforts.

### 4.6. Expanding Prevention in Health Promotion

DOHaD’s primary focus seeks to address unwanted outcomes, ideally well before a disease condition begins to take place. Such efforts are referred to as health promotion and disease prevention. Traditionally, preventing a disease or reducing risk before a disease emerges is referred to as primary prevention, which is sometimes described using an upstream metaphor that emphasizes structural or social factors that reduce risk (income, education, services). Activities or services aimed at reducing or attempting to reverse conditions after disease has initiated is referred to as secondary prevention. Then, those activities or services that seek to help persons manage a chronic disease condition is known as tertiary prevention [41].

DOHaD emphasizes the value of health promotion adopting a form of prevention that precedes primary prevention, known as primordial prevention, because it aims to prevent risk factors from being present by changing social and environmental conditions [33]. Administered before or during human development, primordial prevention seeks to minimize future hazards to health by addressing broad health determinants rather than preventing personal exposure to risk factors, which is the goal of primary prevention. For example, “outlawing alcohol in certain countries would represent primordial prevention, whereas a campaign against drinking [initiation]… would be an example of primary prevention” [41].

Most describe primordial prevention as implementing large-scale (structural) prevention of risk factors (environmental, behavioral, social, economic, etc.) [33]. However, some also define primordial “as prevention of the development of clinical risk factors through the maintenance or adoption of a healthy lifestyle” [42]. Whether at the structural or individual level, primordial prevention seeks to eliminate risk factors outright or replace them with an increasing capacity for protective factors by changing environmental or social determinants of health [42]. Thus, DOHaD provides a strong basis for health promotion to incorporate primordial prevention along with primary, secondary, and tertiary prevention approaches, and presumably enhance its capacity to both promote well-being and prevent disease conditions.

### 4.7. Professional Preparation and Professional Development in Public Health & Health Promotion

A significant part of translating DOHaD into public health functions must involve the consideration of professional preparation. Preparing professionals for public health work not only affects newly trained students of public health, but extends to professionals currently in practice. Because population health and health promotion have vital influence on the environments of human development [2,9], such professional preparation for health promotion and public health should uniquely contribute to DOHaD. For example, an aim for professional development could be on the production of health. The production of health means more than common prevention activities such as disease treatment or disease risk reduction because it also focuses on health promotion, including the strengthening of protective factors. Clearly, this focus values the importance of diagnosing and treating illness, but it also includes an ecologic importance of promoting conditions to support individuals, familial networks, and community partnerships to enhance health. Thus, professional preparation should consider a comprehensive view of disease prevention, health promotion, and clinical interactions through knowledge, skills, and abilities. Disease prevention is often most effective when early disease risk factors are reduced during childhood, before disease onset [43]. This shows that professionals could increase the effectiveness of their interventions by valuing the differences between treating and preventing disease.

Professional preparation should be approached with a unique perspective toward prevention efforts aimed across the life course [9]. For example, rethinking the typical categorization or classification of the causes or consequences of disease, risk, and protective factors including the categorization of psychosocial problems [26] is important because of DOHaD.

Learning from other disciplines and countries (particularly the United Kingdom) can help health promotion and public health embrace strong professional preparation materials [15]. Further, there is likely need to consider new competencies (or reconsider existing competencies) that emphasize SDH [14]. Inherent in DOHaD training materials is the need to value cross-cutting disciplines, including their varying skills and topics. It may mean working collaboratively with clinical practitioners, parents, social workers, teachers, coaches, civic leaders, policy makers, and other invested stakeholders to influence services [26]. Naturally, public health should add its unique lens to such development, but should also reflect value from other perspectives.

## 5. DOHaD Policy Recommendations Relating to Public Health and Health Promotion

### 5.1. Policy-Driven Population Priorities

The World Health Organization (WHO) identified that DOHaD has health implications for millions of children and adults [23]. The application of these implications through policy requires identification of optimal contexts by understanding populations’ past, present, and future needs. As the needs of each population are understood, DOHaD theory can be more effectively applied in interventions to improve health. Identifying certain populations by various methods will clarify the most effective ways to implement DOHaD-based interventions. For example, there may be a need to reconsider the description of populations beyond the characteristics of geography, politics, physical location, gender, or socioeconomic status. Particularly for socioeconomically distinguished populations, DOHaD-based interventions should reflect their context-specific needs. They may include postnatal diets and exercise habits for a developed nation, but in developing nations, focusing on providing sufficient nutrition for pregnant women may be more effective [5]. On the other hand, even though both developed and developing societies may demonstrate sizable differences in culture, economy, etc., both will still likely experience substantial effects as they are relieved from the burdens of NCDs [28]. This shows that DOHaD may have a broad potential to benefit many populations and that the method of intervention should consider specific population needs.

As population needs are researched and identified, health inequities become increasingly apparent and provide potential targets for greatest impact. These findings provide useful direction for public health policy intervention. As NCDs account for 35 million deaths (60% of deaths globally) per year, and around 80% of those are in low- and middle-income countries [7], application of DOHaD research seems to have substantial potential for health improvement in such populations. Furthermore, other potential populations that could be most impacted are the educationally, socially, and economically disadvantaged, as they commonly have a stronger association with illness and a death risk about 80% higher than the general population [44]. Even though several high-income countries focus on “tackling” such health inequities [45], funding for NCD relief efforts in low- and middle-income countries is still disproportionately low based on the disability-adjusted life-year burden of NCDs [7]. This indicates that there could be more potential for improvement by strengthening coordination between populations and reallocating resources to benefit aforementioned populations with the greatest NCD burden.

Other populations of priority include mothers and children. Women with low levels of food security are more likely to give birth to low-weight infants with decreased potential for academic performance, who, because of their hindered development, may in turn perpetuate a similarly insecure environment for their future offspring [23]. Furthermore, low socioeconomic status early on has a greater effect on women’s health compared to men [46]. These findings suggest that women and children are key leverage points to disrupt cycles of poor development and of promoting health optimization for those with the greatest need. Further, focusing such applications especially on girls’ development has been found to be another critical point for economic and social development [7]. By supporting maternal and child health from a DOHaD perspective, and collaborating with agents that affect their life structures and environments, health policy will likely help produce the best continuing health benefits [25].

Structural investment priorities may also need to be considered to better support parents’ capacity, and that do not outsource the parents from providing their full capacity. “The U.S. focus is primarily on funding pre-k education rather than improving parenting ($25 to $1)” [13]. These authors, however, are quick to point out that some parental circumstances or characteristics may be at enough risk that increased non-parental care may be helpful.

### 5.2. Creating Political Will for DOHaD

Many professions point toward public health’s potential additive strength by creating political will for DOHaD through its emphasis on environmental and structural (systemic) approaches [21]. Such structural factors recognize broad influences on health and are closely related to the SDH because they seek to get at the root of the problem. They add credence to policy initiatives such as Health in All Policies (HIAP). Focusing on community strategies that address how environments directly impact human health will assist in creating political will [11]. From a policy perspective, public health’s natural inclination and experience may hasten political will for DOHaD and strengthen its own functional capacity in SDH, HIAP, population health, and place-based strategies.

Because there has been little translation of DOHaD to the public health profession (including key decision makers who influence lawmakers, service providers, and others), there is a great need to build awareness. Simply stated, there is a need to “create awareness of the critical role of early life conditions in the development of health and disease, (with a focus on the links to economic inequality)” [2]. Part of building awareness is the proper dissemination of evidence, whether for policy or other intervention types.

A natural component of political will relates to the allocation of funding. As identified by DOHaD, early investments tend to produce better adult outcomes, and are often more effective in producing positive health outcomes than efforts to change individual health related behaviors [2]. For example, nations that invest the most federal money in the first year of life show superior health improvements, while others who spend more on health later in life demonstrate constrained health improvement [2] given the finite resources available.

### 5.3. Policy Advocacy

Not all public health driven policies are in congruence with DOHaD, and some may need to be modified in order to more adequately reflect disease prevention. For example, the highly touted policies associated with the WHO Millennium Development Goals (MDGs) omitted NCDs and did not consider issues such as unhealthy diet and body composition before and during pregnancy, drug and alcohol abuse in pregnancy, and inadequate breastfeeding practices [6,9]. As a result, there may be a need to reflect DOHaD components among other guidance-based policies or standards including Sustainable Development Goals, Healthy People 2030 (anticipated release by USHHS on or before 2030) and others. Additionally, some policymakers have responded to these findings by implementing policies with unintended consequences. For example, attempts to promote fetal health by prosecuting pregnant mothers with serious substance abuse problems inhibited these mothers from seeking prenatal care due to fear of prosecution [37]. Thus, public health policy regarding maternal responsibilities and rights should view “maternal-fetal rights and health as complementary [which] may allow for more effective legal policies and interventions aimed at improving fetal health trajectories” [37].

Ideal public health policies incorporating DOHaD are likely aimed at primordial and primary prevention to prevent and decrease risk factors and increase protective factors. Examples include provisions that target SDH relating to access to nutritious food, a clean environment, and high quality pre-conception and prenatal care [15]. Wallack and Thornburg [1] point out existing public health related policies that currently improve prenatal and postnatal situations of families. They cite the example of paid parental leave, which is said to impact current and future generations because it prioritizes the urgency of family support.

Public health policy advocates should also represent the needs of the very young, who are unable to voice their opinion using data that reflect their needs. Though likely well-intentioned, an over-reliance on public opinion about those unable to speak for themselves may be less than helpful in promoting DOHaD policies and political good will [7].

### 5.4. Evidence-to-Policy Cycle

Policy driven evidence from longitudinal birth cohorts are needed to develop policies for specific interventions and practices, including economic considerations for cross-generational investments [22,25,31,47]. Such expanding evidence may influence how health is defined and how health care practices are considered [25]. Evidence about the determinants of health/disease trajectories should be examined over wide social and environmental conditions [22]. Doing so may address some of the longstanding gaps in knowledge of chronic disease, and may also provide insight into studies with inconclusive, contradictory, or controversial findings (e.g., the role of exercise or diet in obesity, causes of autism, or factors associated with mental health issues) [20].

## 6. Systemic Coordination across Settings/Players for DOHaD

To consider the translation of DOHaD to public health and health promotion practice, assembling appropriate settings and players is a priority for future planning.

### 6.1. Recommended Players across the Systems

The vulnerable or at-risk persons or groups that may warrant key attention for needs assessment and intervention planning purposes are often those whose various parental patterns may allow, create or foster environments where “chronic exposure to maltreatment [may be] most damaging to developmental health” [38]. While many point to the need to support these and other vulnerable populations, most are also quick to cite the importance of coordinating with multiple community or familial partners. For example, coordination between parties interested in household connections is needed, such as community experts, governmental sectors, NGOs, economic development sectors (including employment), public health entities, clinical sectors (including medicine, social work, etc.), private industry, and retailers, consumer advocates, preschools, recreation centers, religious institutions, and universities, all with an interest in household connections [2,23,26,48]. These types of multi-level coordination are not unfamiliar to public health, but the reasons for coordination may be strengthened as funding providers endorse such work as part of their interests in DOHaD. Aligning financial incentives and coordination are important if public health and health promotion functions are to make significant strides relating to DOHaD.

### 6.2. Example: Cross-Sectoral DOHaD Players in Thailand

Improvements in health of the populations of Thailand have been relatively rapid, enduring, and set an example that may help guide policy makers and implementers to effectively apply DOHaD implications.

Patcharanarumol and colleagues [49] reported that, to surpass the MDGs in 2000, the synergy of many diverse key players in Thailand were essential. These participants included public health workers at every level, a variety of influential Ministry of Public Health personnel (directors-general to local managers), influential technocrats (civil and military), the economic elite, multiple NGOs, and professional groups. These cross-sectorial participants worked collaboratively towards better health of the Thailand population. To produce a significant health reformation, they exercised influence in politics, research, public engagement, education, social equity, public infrastructure, agriculture, transportation, and employment.

By involving these players and working towards their shared vision, Thailand public health workers performed vital roles to form and implement effective networks and policies with strategic vision. Now, as Thailand has made significant improvements in their health system policy formation and implementation, they are strengthening efforts to address SDH to reduce rates of chronic and NCDs. This example, along with many other similar examples, could be useful to guide public health workers in considering collaboration with certain key players to most effectively improve health through applying DOHaD implications in their own contexts.

### 6.3. Recommended Settings across the Systems

Given the players noted above, it is of no surprise that the settings of practice for effective DOHaD translation include family-based interventions and a host of associated areas of public health, community, and clinical practice. Specifically concerning the family setting, there has been a lack of family-based interventions for healthy children, thus making it difficult to compare against interventions for less healthy children. When planning appropriate settings for community or family-based interventions, practitioners should consider interventions at different stages in the life cycle to be population and context-dependent [43]. For example, “[i]t may well be that while a focus on postnatal diet and exercise remains the most promising approach in the developed world, a focus on the health of women and their pregnancies would have greater impact in the developing world” [5].

Clinical settings of practice also note the value of the pediatric medical home model. Such model settings may “serve as a focal point for the reduction of toxic stress and for the support of child and family resiliency” [26]. However, “the pediatric medical home has become the focus of both increasing expectations and formidable challenges” [26]. Challenges include (1) inadequate training for healthcare providers on excessive stress effects; (2) the constraints of office-based approaches; (3) limited accessibility of evidence-based strategies; and (4) financial limitations of the incorporation of such strategies [26].

Regardless of the health development model used to positively impact life course outcomes, most scholars identify that intervention settings should focus less on body systems and individual conditions and focus more toward whole-person, whole-family, and whole-community interests [11]. This approach is expected given a greater emphasis on the SDH [11]. For example, settings that include intermediary determinants (using environment or geographic-based initiatives) and structural levels (upstream social, political, economic) are expected to “produce significant improvements in health, contribute to long-term social change, or improve health equity” [21]. These settings could be planned to improve issues such as healthy eating, tobacco consumption, and active living through environment-based initiatives while also addressing the structural mechanisms that produce unhealthy environments that might otherwise remain untouched.

### 6.4. Planning System-Focused Public Health Interventions

Interventions that support the path to health should reflect an interest in early life conditions and consider the source determinants that promote childhood well-being, which in turn will further their health as adults [2]. However, neither current medical nor public health practices are sufficient to produce optimal health because there is a need to shift toward DOHaD-based programs and policies at a variety of levels across multiple time periods [11]. This will involve basing interventions on research findings and emerging best practices.

### 6.5. Emerging Best Practice Considerations for Intervention

Along with research recommendations, several emerging intervention best practices were recommended for public health’s expanded roles in DOHaD. We will first reflect the priority populations for intervention and will also describe several recommended intervention strategies or methods.

Perhaps the most recommended demographic for interventions are parents with interest in their current and future children [2]. The recommended aim for these interventions is to maximize parents’ capacity to promote an effective developmental environment but also to prevent advertent or inadvertent child maltreatment [38]. One significant challenge to enhancing parental capacity through intervention is the lack of conclusive evidence [38]. However, among those shown to exhibit promise is the pediatric medical home model which has helped mitigate toxic stress and its adverse consequences in the lives of young children [26]. Components of successful medical home models have included the integration of resilience, optimism, commercial programs (such as Reach Out and Read), emotional coaching and parenting programs (such as Incredible Years), home visiting, and several others [26]. When parents or parental figures and other caregivers are supported and trained, evidence suggests that child health outcomes can be improved.

As supported by one or more ecologic frameworks for public health practice, interventions beyond the parental level should include the broader family. For example, Tercyak and Tyc [43] found that family-based programs are among the most effective approaches at preventing excess weight in adulthood. While scholars recommend family interventions, little documentation exists about these steps. Public health and health promotion practitioners should lead out with interventions that support many varieties of family forms.

Almost every scholar in this review pointed to the need for policy, including policy advocacy to promote beneficial interventions. An example of ideal policy may be policy as a passive measure to positively affect at-risk groups. For example, maternal smoking can be reduced while not infringing on maternal autonomy through tobacco control spending, tobacco excise tax increases, and smoking bans, which have been shown to be effective [37].

Finally, interventions should involve widespread dissemination of evidence, stakeholder engagement, and advocacy, because stakeholder support must complement sound and objective application of DOHaD [50].

## 7. Conclusions and Future Directions

In this paper, we have presented implications of DOHaD on public health interests pertaining to population health, SDH, health promotion, health policy, and health systems. Through these discussions, we are optimistic that public health’s direct impact on health promotion and disease prevention will be enhanced using DOHaD principles, research questions, and frameworks across the life course.

First, DOHaD has significant prevention implications for the health promotion and public health professions. Prevention is widely accepted to be both economically and physiologically more effective and beneficial than curative action. DOHaD may provide increased opportunity for public health to promote prevention among all population sectors given the opportunity to focus attention on healthy childhood development with continued supports throughout adulthood. To do so, public health decision makers must give greater weight to the factors that promote early and lifelong human development.

The incentives of prevention may differ depending on the stakeholders involved. This requires more communication and collaboration among sectors to better embrace the shared meaning of prevention. Such prevention challenges require public health functions and collaboration with various sectors of society to implement DOHaD against NCDs with an additional aim to prevent and reduce risk factors. Using DOHaD with an expanded view of prevention, public health with its partners and other stakeholders may anticipate significant improvements in developmental health.

Primary prevention helps to build and maintain health, and will be increasingly focused on upstream conditions to advance all forms of prevention (primordial, primary, secondary, tertiary). Future public health challenges like chronic disease epidemics will outpace remedial efforts if public health workers continue to overemphasize interventions focused on immediate environments, self-determined lifestyles and curative medicine. Therefore, public health functions should (1) strengthen focus on structural and environmental interventions to improve early life; and (2) identify key cross-professional collaboration for NCD prevention, using epigenetic profiling/determinants as guidelines. Along that line, one of several research opportunities includes cross-walking the integration of DOHaD with the 10 Essential Public Health Services.

Public health research for prevention should collaboratively assess biological, environmental, and behavioral indicators during the prenatal process, and then longitudinally assess those conditions over the life course. Such evidence will build the case to more strongly embrace the value of primordial prevention, which seeks to eliminate risk factors outright by changing environmental or SDH. Emerging funding streams will be an important element to drive this research need. The results of this public health research could be used to further develop a conceptual model that illustrates DOHaD’s influence in prevention. Such research could also test the profession’s assumptions regarding the relative impact of individual lifestyles and behaviors on health over the life course. The results of this research could help address a frequent concern raised by select policy makers who insist on seeing the proof of prevention.

Second, public health’s long-held tenet, population health is further supported through DOHaD. An important area to expand capacity for population health by investing more in early life, and by increasing support for disadvantaged populations who have the greatest NCD burdens. For example, programs and policies have begun helping populations, especially women and girls, who represent the most powerful leverage points to break the vicious cycles of hindered development. As a result, more multi-sector collaboration is needed to identify general disease causal pathways and opportunities to incorporate the holistic perspective of DOHaD and ameliorate them.

The increasing value of population health is anticipated to grow with DOHaD, particularly since clinical service providers and health care systems have recently taken great interest in population health [25,51]. As such, DOHaD may become an example of a unity-building approach between medicine and public health to more effectively come together to meet broad population needs.

In that light, future opportunities to expand population health are needed since this term has different meaning between primary care, health system, and public health circles. A DOHaD framework may provide such entities with outlines of necessary social, environmental, and personal factors to move toward a more unified understanding and vision to improve health outcomes.

Third, SDH are more completely understood with DOHaD and may be helpful for public health to build traction to advance HIAP. DOHaD serves as a reminder that the social environment has a profound impact on gene expression and health outcomes, particularly as it is experienced during early developmental stages. These determinants focus on social, environmental, and economic factors that have the greatest influence on health across a community or population.

To move forward, additional SDH should be evaluated using a DOHaD framework (e.g., paternal/maternal health, familial support, prenatal services, etc.). To do that, local and regional data are essential, and should be addressed by interprofessional research and practice teams. For example, clinical and public health players may coordinate using tools such as GIS or shared data access portals to see a spectrum of determinants that are informed by the life course. Such coordination should also lead toward collaborative community engagement that helps consumers, providers, practitioners, and policymakers embrace action guided by SDH and DOHaD. Such interventions may reduce social inequities most powerfully as they focus on critical developmental time periods, such as prenatal through early childhood.

Fourth, health promotion has an important role to advance DOHaD. For example, in addition to providing community programs that promote the prevention of developmental diseases and conditions, health promotion has great capacity to build political will by identifying the value of DOHaD through various strategies such as communication, education, and advocacy. Such efforts are needed so that funding priorities can be aligned with DOHaD’s interests in early life development and life course perspectives. Health promotion can further build this political will by strengthening its current and upcoming workforce through DOHaD enriched professional development and professional preparation.

Another critical health promotion platform should focus on chronic disease prevention by emphasizing the upstream factors affecting early development and well-being. The process of this focus could be termed the production of health. Framed with primordial prevention, such efforts will need to emphasize health and healthy conditions over the life course. Further, healthy conditions should also emphasize the value of risk reduction and prevention. Specific health promotion services and activities should advance these protective and risk reduction efforts for adolescent girls and women concerning nutrition, exercise, weight management, emotional health, and social injustice and advocacy. It will also need to support a culture of health and prevention in workplaces, homes, schools, community organizations, and in relevant public policy. A final area for research could identify measures of well-being to promote health in addition to long-standing measures of preventing ill-health.

Fifth, the great opportunities afforded by DOHaD are certain to come, in part, through health policy. Public health is primed to help take the next steps to integrate DOHaD because of its inherent functions and experiences with health policy reform and advocacy. New policies will be needed and should be informed by diverse stakeholder input. Additionally, some deficiencies in existing policies may need attention. For example, there is a need to consider new standards for Healthy People 2030, Millennium Development Goals, and should include a review of current medical/healthcare (funding and service alignment in the Affordable Care Act) and social (e.g., paid parental leave) programs in the context of public health funding, provision of services, and a common vision to leverage wise policy.

Policies to enhance funding of upstream initiatives for DOHaD could learn from similar initiatives such as Social Impact Bonds and Healthy Futures funding, while also considering economic policies that may limit positive upstream opportunities (e.g., restrict cash/check advance loans, etc.). Supporting SDH and HIAP through health, environmental, and socially-minded policies are examples of shaping environments and social structures for priority populations who need the positive impact of DOHaD research implementation. The policy allocation decisions ahead will be controversial and will require debate. However, DOHaD provides important perspectives from a variety of professionals that will have important public health outcomes.

Coordination among many sectors will need to consider the compiled policy suggestions identified in this paper, and will involve prioritization and partnerships. Throughout coordination, professionals should first seek to obtain data, then share these data to gain societal and political will to form and evaluate ongoing policy for DOHaD.

Sixth, health system thinking is likewise promoted by embracing DOHaD’s role in public health. Aspects such as shared vision, stakeholder engagement, and cross-sector and interdisciplinary collaboration are a few of the inherent tools needed to apply DOHaD for health system strengthening.

While collaboration is important, committed and unified cooperation is much better and should be the goal to move ahead with DOHaD. An integrated health system has a strong commitment and partnership that includes clinical and public health, community organizations and leaders, and local household interests.

Emerging plans for health system/intersystem collaboration will become available over time. One such example with national appeal is the Practical Playbook, which seeks to promote national and local level principles for collaboration, including involvement of the population and all other stakeholders.

The adage, “use funders as conveners” is relevant to the discussion of interdisciplinary collaboration. However, shared data and data sources across many sectors will also reveal important leverage points around which stakeholders can convene and strategize. Using funding and data (among others) as “drivers” or “pain points” may help public health systems to create vision-building opportunities with the potential to not only foster collaboration, but most importantly to align decisions and priorities.

In summary, professional dialogue about the challenges and opportunities for prevention through DOHaD research implementation is important for public health and health promotion. Perhaps the best way to innovate with DOHaD is to build upon and question the basic assumptions under which the profession operates.
